# Bibliometric analysis of traditional Chinese medicine for viral infections through immune modulation (2015–2025)

**DOI:** 10.3389/fimmu.2025.1647900

**Published:** 2025-09-26

**Authors:** Lei Zhang, Shuang Jin, Chuang Qin, DaBao Ma, JinSheng Ye, QingQuan Liu

**Affiliations:** ^1^ Department of Emergency, Yanqing Hospital of Beijing Chinese Medicine Hospital, Beijing, China; ^2^ Capital Medical University, Beijing, China; ^3^ Department of Surgery, Yanqing Hospital of Beijing Chinese Medicine Hospital, Beijing, China; ^4^ Department of Pharmacy, Yanqing Hospital of Beijing Chinese Medicine Hospital, Beijing, China; ^5^ Department of Oncology, Yanqing Hospital of Beijing Chinese Medicine Hospital, Beijing, China; ^6^ Department of General Surgery/Oncology, Beijing Hospital of Traditional Chinese Medicine, Capital Medical University, Beijing, China; ^7^ Yanqing Hospital of Beijing Chinese Medicine Hospital, Capital Medical University, Beijing, China; ^8^ Laboratory for Clinical Medicine, Capital Medical University, Beijing, China

**Keywords:** traditional Chinese medicine, immunomodulation, viral infections, bibliometric, anti-viral, vaccines, cytokine storm

## Abstract

**Objectives:**

The immunomodulatory properties of traditional Chinese medicine (TCM) have attracted significant attention as a strategy for addressing viral infections. However, a comprehensive bibliometric analysis is still lacking. This study aims to systematically identify research trends, knowledge hotspots, and emerging themes in TCM applications for viral infections through immune modulation from 2015 to 2025.

**Methods:**

We collected publications from the Web of Science database from 2015 to 2025 and performed a comprehensive analysis using R, VOSviewer, and CiteSpace. In addition, clinical trial records published during this period were obtained from the PubMed database to assess clinical advancements in this field.

**Results:**

A total of 3,370 publications were analyzed in this study. Between 2015 and 2021, the number of publications in this field showed two distinct stepwise increases, separated by a period of relative stability, followed by a modest decline from 2021 to 2025. China contributed the highest volume of publications and demonstrated the broadest international collaborations, establishing itself as the leading country in this area. Frontiers in Immunology published the largest number of articles, while the Journal of Virology was the most frequently cited journal. Core topics included “Infection,” “COVID-19,” “Expression,” “Antiviral,” and “Protein.” The primary research focus centered on TCM’s antiviral effects and its modulation of immune responses, investigating its regulatory impact on inflammation and cytokine storms during viral infections, and examining TCM’s role in modulating immune responses to viral vaccines. Clinical trials in this field focus on improving the management of viral infections, and immune reconstitution strategies for chronic infections.

**Conclusion:**

This study systematically analyzes the scientific literature in this field, providing valuable insights into current research trends and highlighting future directions in the application of TCM to the immunomodulation of viral infections.

## 1 Introduction

Viral infections pose a significant challenge to global public health, substantially contributing to worldwide morbidity and mortality. Notable viruses such as severe acute respiratory syndrome coronavirus 2 (SARS-CoV-2) (1), influenza viruses (2), hepatitis viruses (3–5), the human immunodeficiency virus (HIV) (6, 7), and poliovirus (8), continue to threaten public health. Currently, about 270 viruses are known to infect humans (9). Worryingly, Viral infections are closely associated with increased mortality; for instance, the global SARS-CoV-2 pandemic caused an estimated 8.8 million deaths in 2021, ranking it as the second leading cause of death after ischemic heart disease. This outbreak also reduced global life expectancy at birth to 71.4 years and healthy life expectancy to 61.9 years, levels last seen in 2012 (1, 10). The devastating impact of viral pandemics extends beyond SARS-CoV-2, with historical outbreaks consistently leading to severe consequences. In response, the international community has prioritized various viral diseases in health-related sustainable development goals, including acquired immune deficiency syndrome (AIDS), hepatitis, and poliomyelitis as core indicators for monitoring progress (10). The prevalence of these outbreaks not only escalates medical and financial challenges but also signifies a transition in the global disease burden towards infectious diseases (1, 2, 10).

Current strategies for combating viral infections fall into three main categories: targeted antiviral drugs, prophylactic vaccines, and supportive care. Despite significant advancements, major challenges persist: (1) the specificity, resistance, and timing issues of antiviral agents (11, 12). (2) vaccines’ limited efficacy against variant strains (13, 14). (3) The absence of efficient therapeutic options for particular viruses, such as respiratory syncytial virus (15) and emerging pathogens. These limitations severely constrain clinical management. Encouragingly, high-quality clinical research increasingly supports the efficacy of traditional Chinese medicine (TCM) in treating viral infections (16–18).

Over a century ago, Canadian internist Sir William Osler observed that mortality from infections is primarily due to the host’s response rather than the pathogen itself (19). Immune dysregulation subsequent to viral infection remains a key factor in severe complications (20). While the concept of “immunity” was officially defined in Chinese medical literature during the Ming Dynasty in the *Mian Yi Lei Fang*, the connection between infectious diseases and the immune response was already suggested in the pre-Qin period. The *Yellow Emperor’s Canon of Internal Medicine* examines the transmission during epidemic outbreaks, whereas the *Treatise on Cold Damage Diseases* presents a systematic account of the progression of externally contracted diseases, both providing early insights into the relationship between immunity and infectious diseases.

The immune protection conferred by TCM against viral infections is primarily achieved through the bidirectional regulation of innate and adaptive immune responses (21). By modulating Toll-like receptors (TLRs), natural killer (NK) cells, and the functions of neutrophils and macrophages, the active components of TCM offer a robust strategy for addressing viral infections within innate immunity (22–24). Additionally, these Chinese herbal compounds are pivotal in adaptive immunity, promoting immune homeostasis and recovery by regulating T cells, B cells, and cytokines (22, 24, 25). Despite extensive research on the immunomodulatory effects of TCM in viral infections, analyses of research hotspots and emerging trends are lacking. This gap prevents new researchers from quickly grasping the field and impedes the advancement of TCM immunotherapy for viral infections.

Bibliometric analysis serves as a systematic tool to explore the evolution of academic fields (26). Utilizing extensive databases like Web of Science, it identifies key aspects of a field, including contributing countries, significant publications, research focuses, and collaborative networks (27). Such analyses establish a robust basis for comprehending the progression of knowledge development and anticipating forthcoming research domains (28). Despite growing interest in TCM immune modulation for viral infections, systematic bibliometric studies are scarce, hindering a comprehensive understanding of the field’s evolution. This study analyzes literature from 2015 to 2025 to identify trends, key contributors, main research themes, and emerging frontiers. By addressing this gap, we aim to provide an integrated overview of the field, foster international collaboration, guide future research, and support evidence-based application of TCM immune modulation in antiviral therapy.

## 2 Materials and methods

### 2.1 Data collection

Due to its broad multidisciplinary coverage being included (with over 12,000 high-impact journals being covered) and its comprehensive metadata being made available for citation analysis and collaboration network construction, the Web of Science Core Collection (WoSCC) was chosen as the primary data source for this bibliometric analysis (29). Data were retrieved via Capital Medical University’s institutional subscription on May 4, 2025. After removing irrelevant records, a total of 3,370 eligible publications were identified. In addition, relevant clinical trial data were obtained from the PubMed database, as such information is not available in WoSCC. The search strategies are detailed in Annex 1. Duplicate entries were removed, and the remaining articles were saved in plain text format with cited references exported as complete records. Results of clinical trials were exported in PubMed format.

### 2.2 Data analysis

This study utilized sophisticated data visualization and scientific knowledge mapping tools for the bibliometric analysis, specifically employing Origin 2018, R software (version 4.5.0, http://www.bibliometrix.org) (30), VOSviewer (version 1.6.20) (31), and CiteSpace (version 6.4.R1) (32). National and institutional co-authorship networks, as well as source co-citation and keyword co-occurrence analyses, were visualized using VOSviewer. The specific parameters were as follows: (1) The national co-authorship network included countries with at least 5 publications; (2) The institutional co-authorship network included institutions with at least 13 publications; (3) Source co-citation analysis considered sources with a minimum of 189 citations; (4) Keyword co-occurrence analysis included keywords that appeared at least 23 times, with synonymous terms merged. The impact factors used in this study were obtained from Journal Citation Reports (JCR) for the year 2023.

## 3 Results

### 3.1 General landscapes of global publications

**Figure 1:**
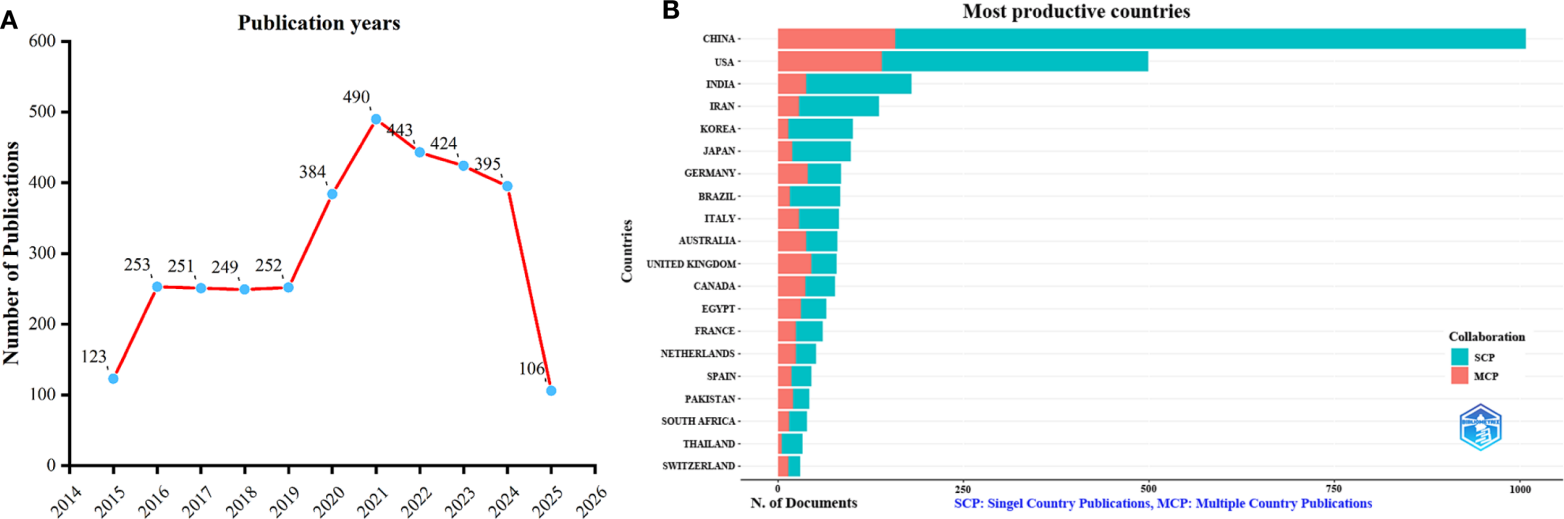
Annual publication trends of traditional Chinese medicine for viral infection through immune modulation, 2015–2025. **(A)** Yearly publication trends. **(B)** Country distribution and international collaboration of corresponding authors.

The dataset consisted of 3,370 publications sourced from WoSCC. As shown in **Figure 1A**, the annual publication count on TCM for viral infections through immune regulation displayed two major growth phases: an initial rise from 2015 to 2016, with an increase of 130 publications (representing 105.6% growth), followed by a more substantial surge between 2019 and 2021, during which output grew by 238 publications (94.4% increase over two years), peaking at 490 articles in 2021. These periods of expansion were separated by a plateau from 2017 to 2019, characterized by minimal annual variation in publication numbers (approximately ±1.6%). After 2021, a gradual decline in annual publications was observed. This trend is closely linked to major global events, including the 2015 Nobel Prize awarded to Professor Tu Youyou (33) and the outbreaks of Zika, Ebola (34, 35), and COVID-19 (36–38), all of which heightened interest in TCM-mediated immune modulation. As the COVID-19 pandemic has stabilized, research activity and publication output in this area have shown a slight decline since 2021.

**Figure 2:**
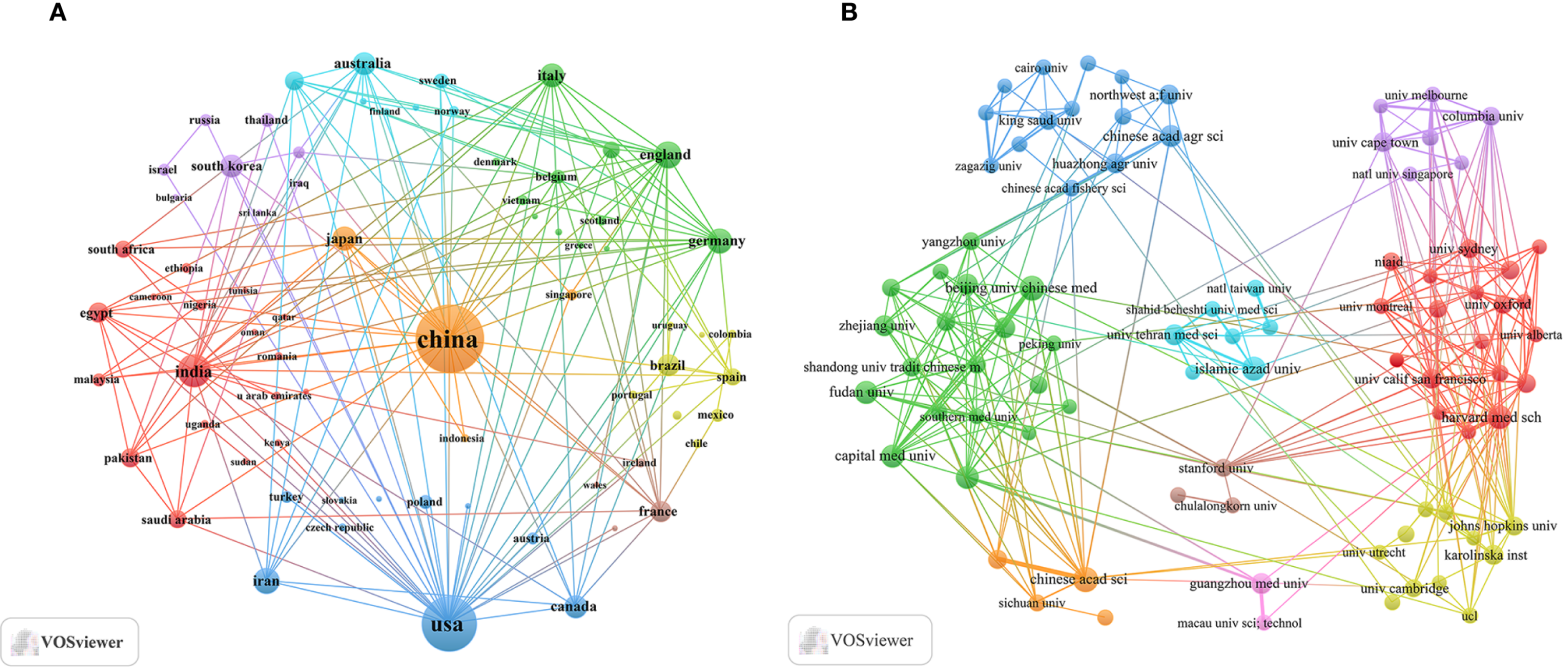
Countries/regions and institutional collaboration maps of traditional Chinese medicine for viral infection through immune modulation, 2015–2025. **(A)** International collaboration map. **(B)** Institutional collaboration map.

An analysis of corresponding authors’ countries showed that China (n = 1008) was the primary contributor, followed by the USA (n = 499), India (n = 180), Iran (n = 136), and Korea (n = 101). Furthermore, as presented in **Figure 1B** and detailed in **Table 1**, 15.7% of publications from China and 28.1% from the USA involved multi-country collaborations (MCPs). Notably, China not only leads in publication volume but also maintains an extensive international collaboration network, as illustrated in **Figure 2A**. Furthermore, the collaboration map reveals that Beijing University of Chinese Medicine (n = 37) and the Chinese Academy of Sciences (n = 37) serve as major hubs of collaboration in this field(**Figure 2B**; **Table 2**). These findings indicate that researchers in China prioritize the exploration of the immunomodulatory effects of TCM concerning viral infections. This trend appears to be influenced by China’s unique context, which encompasses a rich heritage in herbal medicine, strong governmental backing for TCM research, and proactive policies that encourage international scientific collaboration.

**Table 1:** Most relevant countries by corresponding authors.

Countries	Articles	SCP	MCP	Freq	MCP_ratio
China	1008	0.299	850	158	0.157
USA	499	0.148	359	140	0.281
India	180	0.053	142	38	0.211
Iran	136	0.04	108	28	0.206
Korea	101	0.03	87	14	0.139
Japan	98	0.029	79	19	0.194
Germany	85	0.025	45	40	0.471
Brazil	84	0.025	68	16	0.19
Italy	82	0.024	54	28	0.341
Australia	80	0.024	42	38	0.475
United Kingdom	79	0.023	34	45	0.57
Canada	77	0.023	40	37	0.481
Egypt	65	0.019	34	31	0.477
France	60	0.018	36	24	0.4
Netherlands	51	0.015	27	24	0.471
Spain	45	0.013	27	18	0.4
Pakistan	42	0.012	22	20	0.476
South Africa	39	0.012	24	15	0.385
Thailand	33	0.01	28	5	0.152
Switzerland	30	0.009	16	14	0.467

SCP, Single Country Publication; MCP, Multiple Country.

**Table 2:** Most relevant author affiliations in publications on traditional Chinese medicine for viral infection immunoregulation.

Affiliations	Articles
Beijing University Of Chinese Medicine	37
Chinese Academy of Sciences	37
Islamic Azad University	34
Harvard Medical School	33
Capital Medical University	32
Fudan University	31
Sun Yat-sen University	31
Chinese Academy of Agricultural Sciences	29
Guangzhou Medical University	26
Zhejiang University	26
Chengdu University of Traditional Chinese Medicine	25
China Academy of Chinese Medical Sciences	25
University of Tehran Medical Sciences	25
Huazhong Agricultural University	24
Karolinska Institute	24
Northwest A&F University	23
University of California, San Francisco	23
University of Cape Town	23
Shandong University of Traditional Chinese Medicine	22
University of Sydney	22

### 3.2 Journals and co-cited journals

In order to ascertain the journals exhibiting the greatest publication and citation impact within the domain of TCM pertaining to viral infections via immune modulation, we utilized the Bibliometrix package in R software (version 4.5.0). The visualizations were generated utilizing the ggplot2 package. Furthermore, a journal co-citation analysis was conducted utilizing VOSviewer (version 1.6.20).

**Figure 3:**
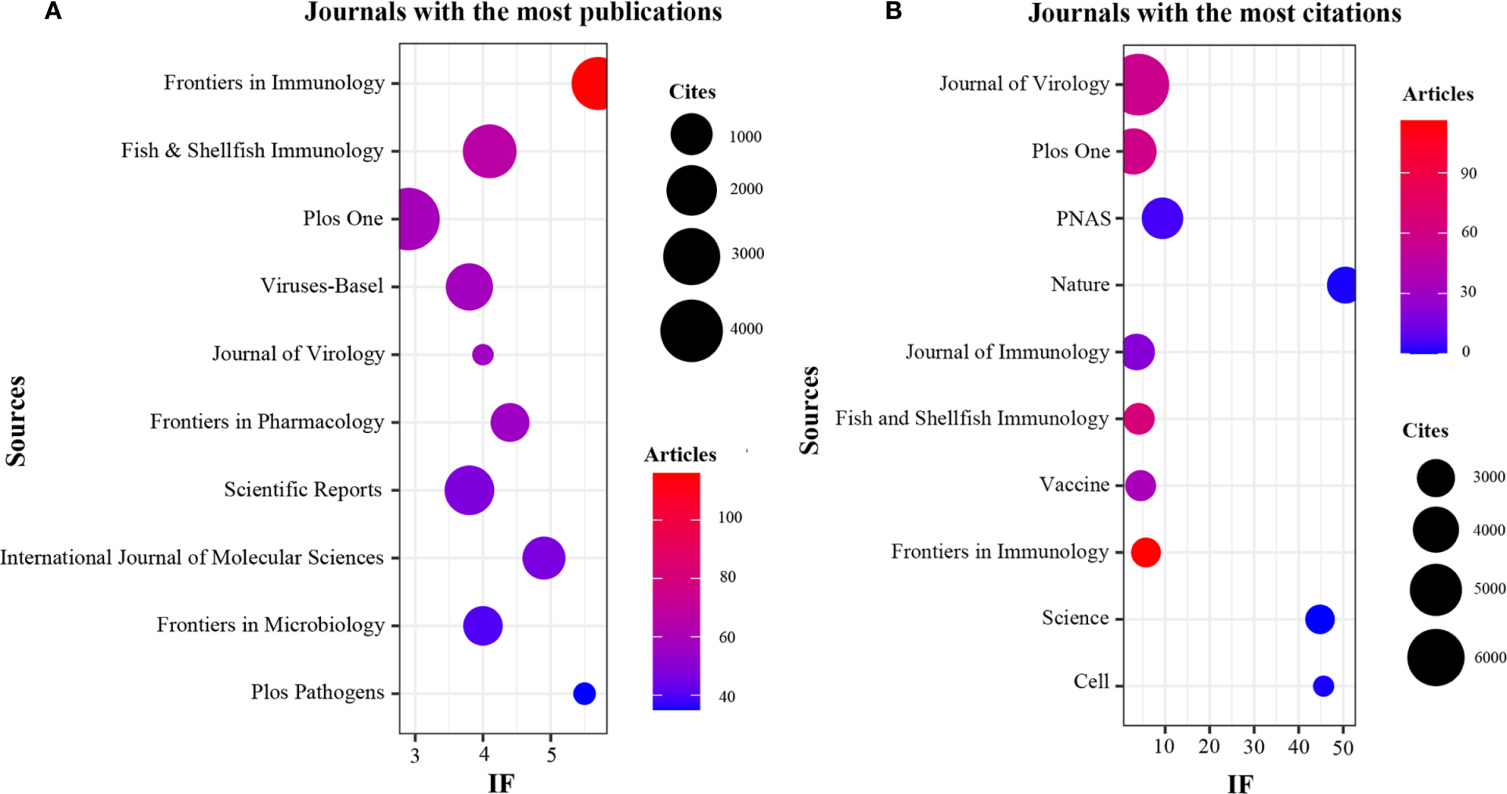
Journals with the most publications and journals with the most citations. **(A)** Journals with the most publications. **(B)** Journals with the most citations.

**Figure 4:**
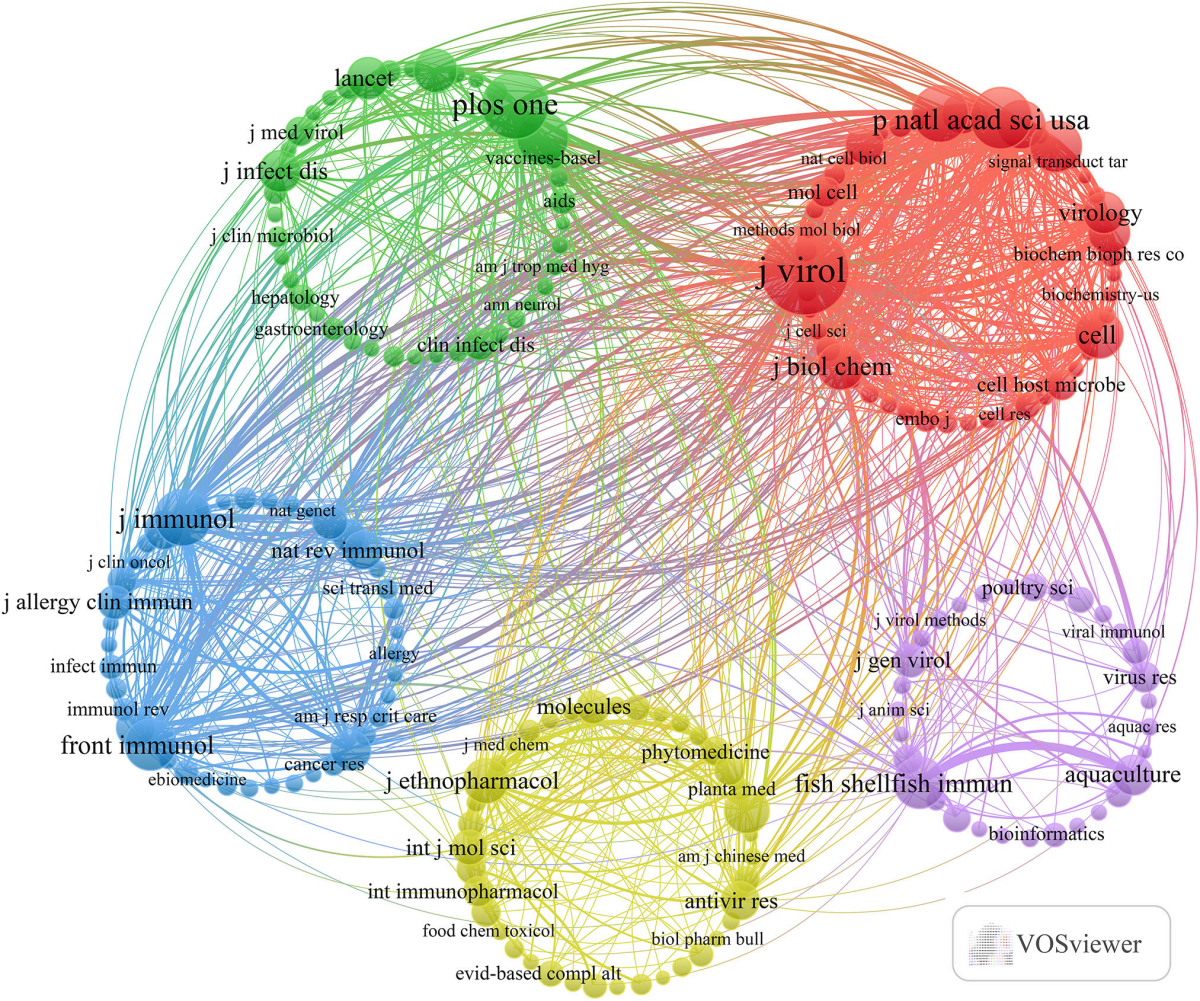
Co-citation journals of traditional Chinese medicine for viral infection through immune modulation.

The present study revealed a comprehensive collection of 3,370 documents distributed among 1126 academic journals (refer to **Supplementary Material S2** for further details). As demonstrated in **Table 3** and represented in **Figure 3A**, Frontiers in Immunology (n = 116, IF = 5.7) has emerged as the predominant publisher, succeeded by Fish & Shellfish Immunology (n = 66, IF = 4.1), PLoS One (n = 59, IF = 2.9), Viruses-Basel (n = 58, IF = 3.8), and Journal of Virology (n = 57, IF = 4.0). **Table 4** and **Figure 3B** present an analysis of the most frequently cited journals, which include the Journal of Virology (n = 6997, IF = 4.0), PLoS One (n = 4002, IF = 2.9), PNAS (n = 3375, IF = 9.4), Nature (n = 2884, IF = 50.5), and the Journal of Immunology (n = 2855, IF = 3.6). It is noteworthy that the co-cited journals map presented in **Figure 4** illustrates that the Journal of Virology, PLoS One, and Frontiers in Immunology serve as pivotal collaboration hubs. The collective findings highlight the significant contribution of the Journal of Virology to the domain of TCM in the treatment of viral infections via immune system modulation.

**Table 3:** Top 10 journals with the most publications.

Sources	Articles	IF (2023)	Citations
Frontiers in Immunology	116	5.7	2374
Fish & Shellfish Immunology	66	4.1	2482
Plos One	59	2.9	4002
Viruses-Basel	58	3.8	1580
Journal of Virology	57	4.0	1
Frontiers in Pharmacology	56	4.4	726
Scientific Reports	48	3.8	1890
International Journal of Molecular Sciences	47	4.9	1108
Frontiers in Microbiology	40	4.0	773
PLoS Pathogens	35	5.5	4

IF, Impact Factor.

**Table 4:** Top 10 journals with the most citations.

Sources	Citations	IF (2023)	Documents
Journal of Virology	6997	4.0	57
Plos One	4002	2.9	59
PNAS	3375	9.4	5
Nature	2884	50.5	1
Journal of Immunology	2855	3.6	21
Fish and Shellfish Immunology	2482	4.1	66
Vaccine	2436	4.5	35
Frontiers in Immunology	2374	5.7	116
Science	2359	44.8	0
Cell	2149	45.6	1

IF, Impact Factor.

### 3.3 Citation burst

**Figure 5:**
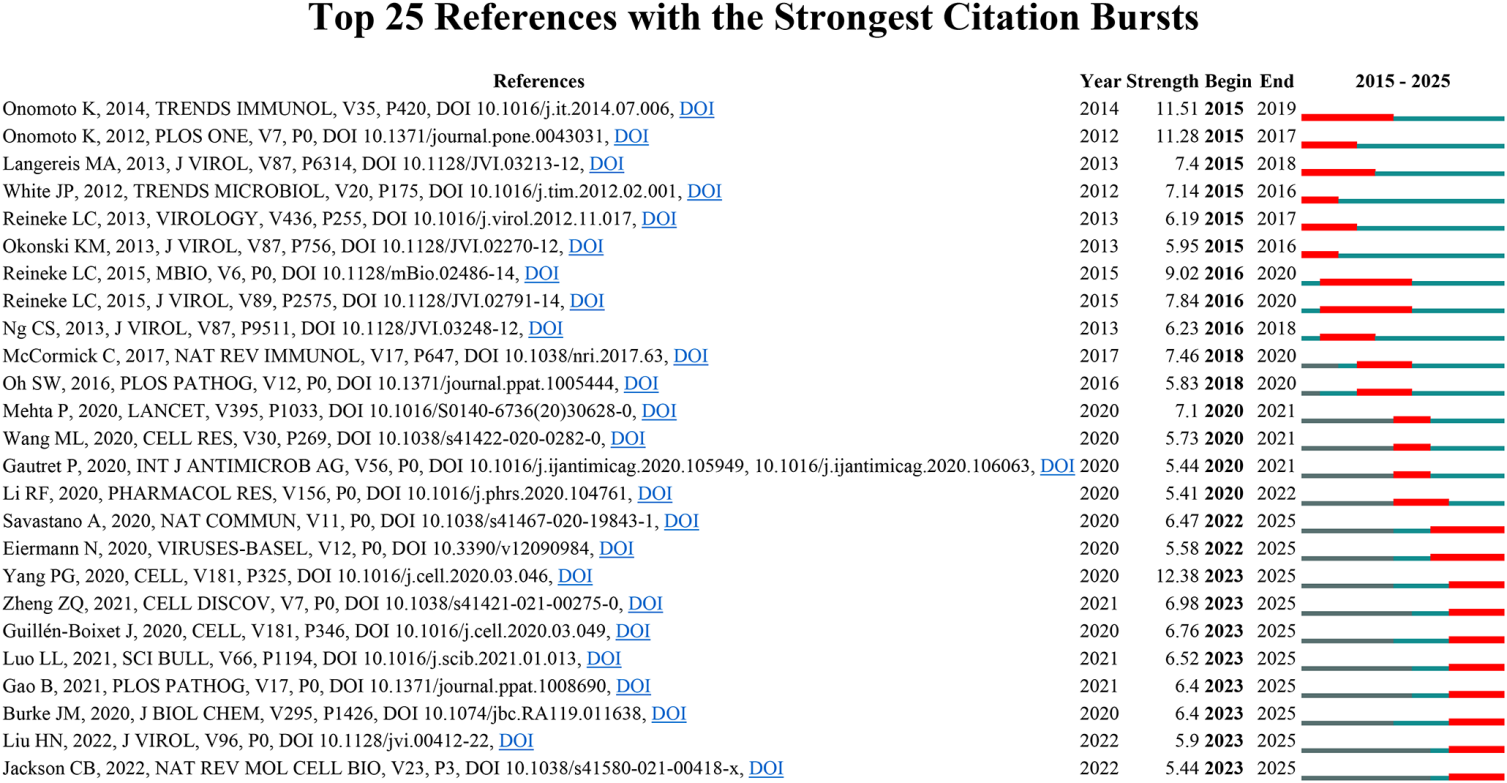
The 25 most cited references on traditional Chinese medicine for viral infection through immune modulation.

In order to conduct a comprehensive examination of the frontier areas and focal points within TCM concerning viral infections through immune modulation, we utilized CiteSpace to identify 127 references exhibiting significant citation bursts according to established criteria (top 25; status count: 2; minimum duration: 2). A selection of 25 references is illustrated in **Figure 5**. The complete compilation of these citations along with their respective DOIs can be found in **Supplementary Material S3**. It is noteworthy that the three references exhibiting the most significant citation bursts were: (1) “G3BP1 Is a Tunable Switch that Triggers Phase Separation to Assemble Stress Granules” (strength: 12.38); (2) “Antiviral innate immunity and stress granule responses” (strength: 11.51); (3) “Critical Role of an Antiviral Stress Granule Containing RIG-I and PKR in Viral Detection and Innate Immunity” (strength: 11.28). Additionally, the three latest emerging citation bursts have been identified as follows: (1) “Mechanisms of SARS-CoV-2 entry into cells”; (2) “SARS-CoV-2 N Protein Antagonizes Stress Granule Assembly and IFN Production by Interacting with G3BPs to Facilitate Viral Replication”; (3) “RNase L promotes the formation of unique ribonucleoprotein granules distinct from stress granules”.

In summary, illustrating from the citation burst analysis, we have discerned three principal research focal points within the domain of TCM concerning viral infections through immune system modulation: (1) The relationship between stress granules and antiviral immune responses, emphasizing the interplay between cellular stress mechanisms and innate immunity; (2) The phenomenon of cytokine storm and immune modulation in viral infections, investigating the role of TCM formulations in regulating inflammatory responses during severe viral illnesses; (3) The mechanisms of antiviral compounds, analyzing the dual action of TCM preparations in direct viral inhibition and the regulation of the immune system.

### 3.4 Keyword clusters and evolution

Keyword cluster analysis serves as a valuable method for pinpointing research hotspots and developmental trends within scholarly disciplines. This research employed VOSviewer software to extract a total of 15,952 keywords from the existing literature. **Table 5** presents a comprehensive analysis of keyword frequency, revealing that 15 terms have been recorded with over 150 occurrences. Notably, “Infection” leads the list with 424 instances, succeeded by “COVID-19” (n=389), “Expression” (n=306), “Antiviral” (n=210), “Protein” (n=204), “*In-Vitro*” (n=203), “Cells” (n=198), and “Activation” (n=195).

**Table 5:** Top 15 keywords related to traditional Chinese medicine for immunoregulation in viral infections.

Rank	Keywords	Counts
1	Infection	424
2	COVID-19	389
3	Expression	306
4	Antiviral	210
5	Protein	204
6	*In-Vitro*	203
7	Cells	198
8	Activation	195
9	Extract	181
10	Vaccine	181
11	Innate Immunity	172
12	Human Immunodeficiency Virus	171
13	Inflammation	166
14	Replication	165
15	Stress Granule	157

**Figure 6:**
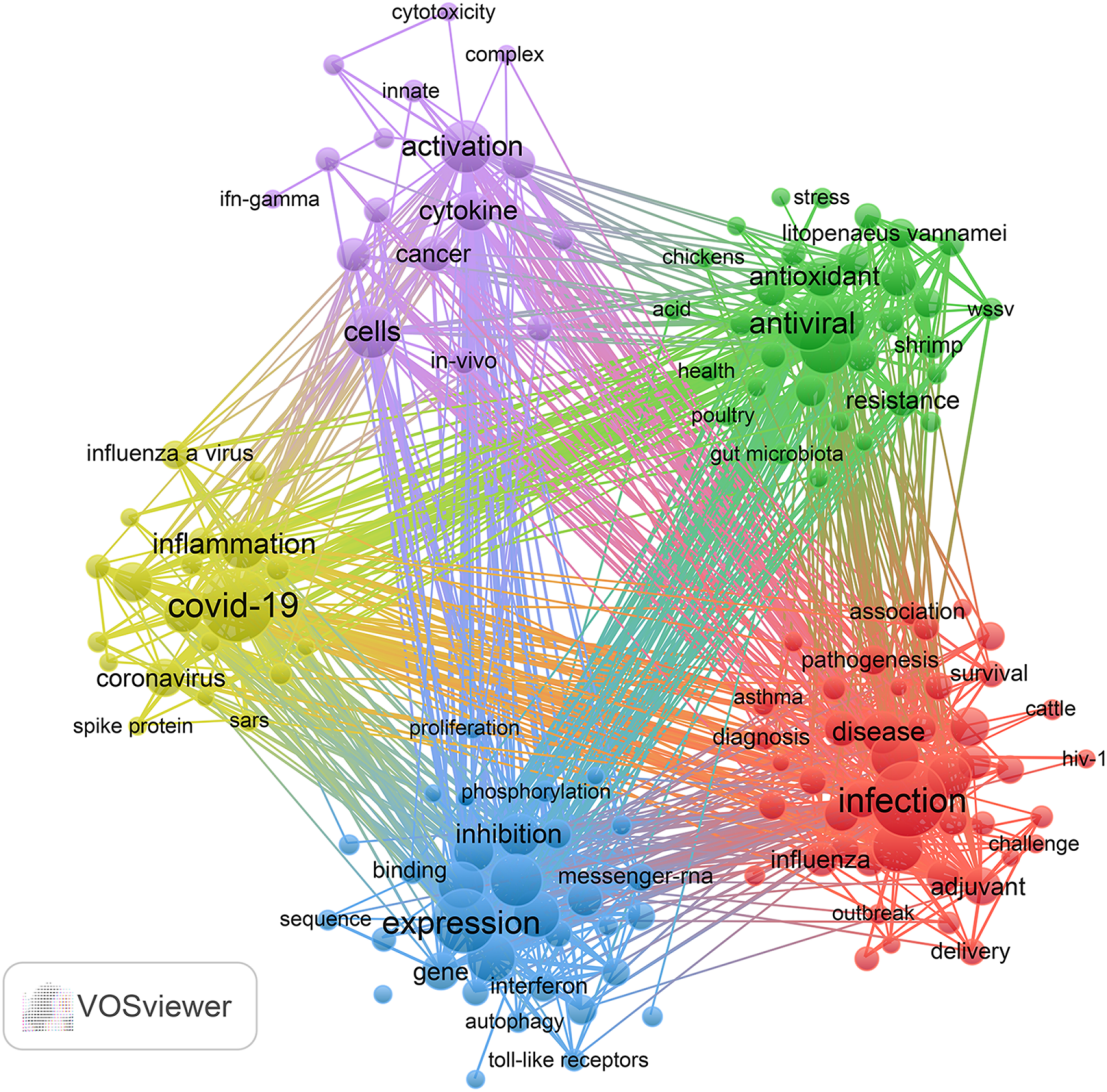
Keyword co-occurrence map of publications on traditional Chinese medicine for viral infection through immune modulation.

Added to that, we pointed out 197 keywords that met a minimum frequency threshold of 23 occurrences, which were subsequently utilized to create a keyword cluster map (**Figure 6**). The map delineates five distinct clusters, each indicated by a unique color. Utilizing cluster analysis, we discerned five unique clusters: (1) Cluster 1 (red dots): This cluster centers on the epidemiological characteristics of viral infections and explores advances in immunization strategies, with particular reference to TCM adjuvant therapies and their influence on vaccine efficacy, antibody generation, and overall immune response. Key terms in this group include infection, vaccine, antibody, adjuvant, and efficacy. (2) Cluster 2 (green dots): This cluster presents research concerning the antiviral activities and underlying molecular mechanisms of traditional medicine-derived compounds. Representative keywords comprise antiviral, extract, antioxidant, gene expression, and resistance, underscoring the therapeutic potential of TCM constituents in combating viral pathogens. (3) Cluster 3 (blue dots): This cluster emphasizes studies focused on the modulation of innate immune responses and host-pathogen interactions, particularly through TCM interventions. Central terms incorporate expression, protein, innate immunity, replication, and interferon, reflecting how traditional medicine may enhance innate defenses against viral invasion. (4) Cluster 4 (yellow dots): This cluster highlights research into immune-mediated inflammatory pathways and the application of multi-targeted pharmacological approaches typical of TCM in respiratory viral infections. Dominant terms in this cluster include COVID-19, inflammation, mechanism, NF-κB, and network pharmacology, showcasing the broad-spectrum regulatory effects of TCM. (5) Cluster 5 (purple dots): This cluster relates to the cellular immune landscape during viral infection and illustrates how TCM-related therapies modulate immune cell activation, cytokine production, and interactions involving dendritic cells, macrophages, and receptors. Representative keywords are cells, activation, cytokine, cancer, dendritic cells, influenza virus, *in-vivo*, receptor, macrophages, and Epstein-Barr virus. The entirety of the keywords encompassed within these five clusters can be found in **Supplementary Material S4**.

**Figure 7:**
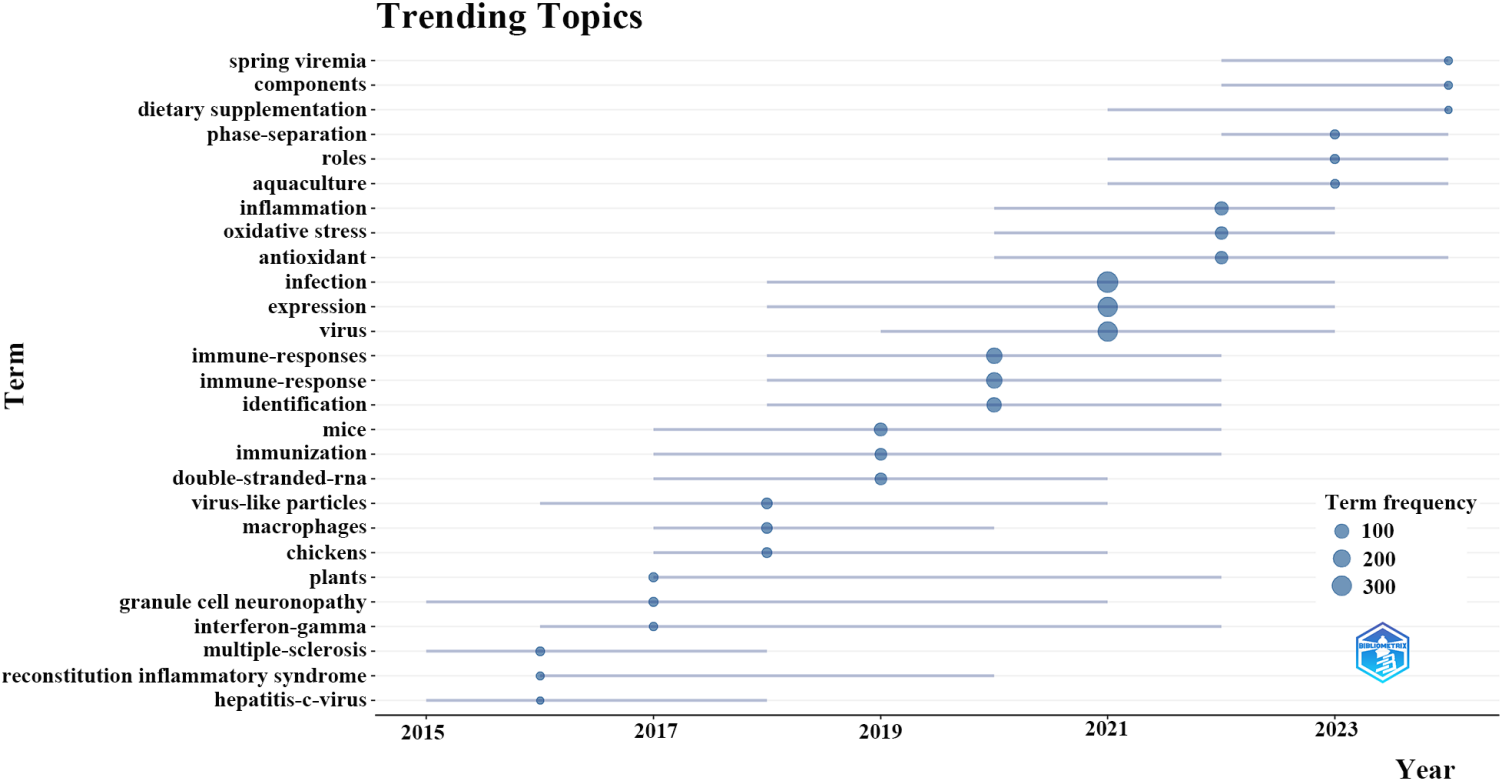
Trending topics of traditional Chinese medicine for viral infection through immune modulation.

Beyond that, we developed a dynamic thematic progression chart applying the bibliometrix toolkit to discern the evolving trends in research (**Figure 7**). The evolution of the field exhibits a distinct chronological advancement: from 2015 to 2017, investigations focused on fundamental viral infection mechanisms, gene expression, and the characterization of basic immune responses. Subsequently, from 2017 to 2019, research transitioned to explore specific immunological processes such as oxidative stress, antioxidant activity, and macrophage function. The period from 2019 to 2021 was markedly shaped by the COVID-19 pandemic, during which there was a notable rise in the focus on inflammation, advanced immune signaling, and network pharmacology methodologies. In the most recent years (2021-2023), investigations have progressed towards practical applications that include dietary supplementation, phase-separation methodologies, and specific environments such as aquaculture. This thematic evolution indicates that forthcoming research will concentrate on clarifying the specific mechanisms of TCM compounds, innovating delivery systems, and substantiating the efficacy of TCM through thorough clinical investigations, especially in the context of emerging viral and immune-related diseases.

### 3.5 Clinical progress analysis

A total of 8 clinical trials were retrieved from the PubMed database (Annex 5). These studies can be broadly categorized into two main research themes: (1) The application of TCM for treating viral infections through immune modulation; (2) TCM as an immune reconstitution strategy for patients with chronic viral infections.

## 4 Discussion

### 4.1 General information

In this study, we analyzed 3,370 publications from 2015 to 2025 using bibliometric and visual methods. The results reveal a growth pattern from 2015 to 2021, with a notable surge in 2016 when publications doubled to 253. Output then stabilized for three years, followed by a rapid increase, peaking at 490 articles in 2021 (238 more than in 2019). Post-2021, the number of publications gradually declined, although it remained above pre-2019 levels. Data for 2025 is incomplete due to the May 4 cutoff.

First of all, the award of the 2015 Nobel Prize in Physiology or Medicine to Professor Tu Youyou for her discovery of artemisinin was a pivotal event that elevated scholarly interest and policy support for TCM research (33). Professor Tu’s work, influenced by historical Chinese medical texts such as the “*Vade Mecum with Prescriptions for All Emergencies*” by Old Immortal Ge, was recognized on the international stage, spurring increased funding and policy initiatives for TCM investigations. Additionally, the emergence of public health crises, such as the Zika and Ebola virus epidemics (34), heightened awareness of the potential role of TCM in viral prevention (35), leading to a significant increase in academic publications on the topic.

In the wake of the global COVID-19 pandemic that emerged in 2020 (36, 37), TCM gained prominence in China’s strategies for managing and mitigating the crisis (38, 39). This highlighted the profile of TCM’s role in modulating immune responses to viral infections, sparking growing global interest in its applications for immune regulation and viral prevention (40). Consequently, research institutions and journals increased support for TCM-related studies, fueling rapid growth in the literature. However, as the COVID-19 pandemic came under control, research enthusiasm waned, leading to fewer large-scale studies and publications. With most initial findings already published, innovation slowed, and research shifted to synthesis, further reducing output. Additionally, diversified funding and priorities have redirected resources to other fields. It is worth noting that the apparent decline in publications observed in 2025 may be due to incomplete data, as the data collection for this study ended on May 4 of that year, providing a technical explanation for the observed downward trend.

China leads globally in scholarly publications on TCM related to viral infections and immune modulation, highlighting significant engagement by Chinese researchers. This focus is driven by TCM’s local prominence and supportive national policies. Furthermore, by utilizing robust research infrastructure and a large number of highly qualified researchers, Beijing University of Chinese Medicine and the Chinese Academy of Sciences have become key collaborative hubs. A total of 3,370 publications appear across 1,126 journals, with Frontiers in Immunology, Fish & Shellfish Immunology, PLoS One, Viruses-Basel, and the Journal of Virology being the most prolific. The Journal of Virology and PLoS One are notably the most cited, serving as central collaborative platforms and establishing themselves as leading journals in this field.

### 4.2 Hotspots and development trends

As described above, a thorough bibliometric analysis has revealed emerging research hotspots related to the application of TCM in viral infection through immune system modulation. The findings indicate that research frontiers and focal areas in this domain mainly concentrate on three themes. First, contemporary research has increasingly focused on delineating the antiviral actions of TCM, not only through direct inhibition of viral replication and entry but also through immunomodulation that encompasses both innate and adaptive immune responses, thereby enhancing host antiviral defenses. Second, substantial effort has been devoted to characterizing the capacity of TCM to regulate inflammation and mitigate cytokine storm during viral infections, highlighting its significance in curbing excessive immune responses and reducing immunopathological consequences. Finally, accumulating evidence indicates that TCM can modulate vaccine-induced immune responses and may function as an adjuvant to enhance vaccine immunogenicity and effectiveness. Collectively, existing studies provide supportive evidence for these applications across therapeutic and preventive contexts.

Expanding upon the fundamental concept that TCM plays a role in antiviral defense through both direct and indirect mechanisms, we initially outline its direct antiviral effects. TCM have demonstrated notable direct antiviral effects through multiple mechanisms targeting different stages of the viral life cycle. Initially, numerous components of TCM impede viral entry by obstructing attachment to host-cell receptors or by inhibiting membrane fusion. For instance, glycyrrhizin and glycyrrhizic acid, which are extracted from licorice, demonstrate a significant ability to obstruct viral adsorption and penetration (41, 42). In a similar vein, the active constituents of Artemisia vulgaris and perilla leaf extracts possess the ability to directly inactivate viral particles or disrupt their interactions with host receptors (43, 44). Secondly, for viruses that have effectively infiltrated cells, constituents of TCM can function by inhibiting viral replication, focusing on essential viral enzymes (such as polymerases) and the production of viral nucleic acids and proteins. The utilization of glycyrrhizic acid–based carbon dots, Lianhua Qingwen Capsule, Huashi Baidu Decoction, baicalin, and associated natural flavonoid derivatives has been shown to diminish viral load and efficiently inhibit replication through various mechanisms (41, 45–47). Additionally, compounds like emodin and dandelion extracts have been shown to downregulate the expression of essential viral genes during post-entry stages, consequently hindering protein synthesis and viral proliferation (48, 49). Furthermore, a significant aspect of direct action involves direct virucidal activity, in which the physical disruption of viral structures results in their inactivation. For example, various herbal extracts obtained from forsythia and honeysuckle have demonstrated the ability to compromise the viral envelope, significantly diminishing infectivity (47). In conclusion, the findings suggest that TCM alleviates viral infection via several direct mechanisms, such as obstructing viral entry, inhibiting replication, compromising structural integrity, and interfering with viral release.

Beyond direct mechanisms, TCM also significantly contributes to the enhancement of the host immune response, which serves as a vital indirect approach in the fight against viral infections. Through the modulation of both innate and adaptive immune mechanisms, TCM enhances the overall immune defense, thereby facilitating more effective viral clearance and bolstering host resistance (21). The innate immune system, the first defense against pathogens, includes macrophages and NK cells. TCM activates pattern recognition receptors to bolster innate immunity (50). Xuanfei Baidu Decoction modulates the PD-1/IL17A pathway, reducing pro-inflammatory cytokine secretion and neutrophil and macrophage infiltration (51, 52). Lianhua Qingwen promotes M2 macrophage infiltration, decreases M1 macrophage markers, and alleviates inflammation in Raw264.7 macrophages, significantly enhancing their phagocytic capacity (53, 54). In H1N1-infected mice, San Wu Huangqin decoction boosted NK cell activity, accelerated the phagocytic function of macrophages (55). Overactive innate immune responses, such as excessive macrophage infiltration post-RSV infection, worsen outcomes like pneumonia and asthma; Xuanfei Fang formula can counteract these effects (56). It is essential to emphasize that research has demonstrated that TRIM29 and PARP9 serve as significant regulators of antiviral immunity. The absence of TRIM29 confers protection against fatal infections caused by influenza virus and other DNA/RNA viruses by augmenting the antiviral innate immune response in alveolar macrophages (57), dendritic cells (58, 59), and intestinal epithelial cells (60). Furthermore, PARP9 functions as a noncanonical sensor for RNA viruses, playing a significant role in the defense against RNA virus infections (61). The incorporation of these mechanisms will deepen the comprehension of TCM-mediated immunomodulation within the framework of viral infection (62).

Furthermore, the adaptive immune system, primarily composed of T and B lymphocytes, is crucial for antigen-specific responses and immunological memory. Astragaloside, the main active component of Astragalus membranaceus, exerts immunoregulatory effects by promoting T-cell activation, balancing effector and regulatory T cells, enhancing CD45 phosphatase activity, and inhibiting pro-inflammatory cytokine production and the NF-κB pathway (63). Beyond astragalus saponin, numerous other Chinese medicines also exhibit immunoregulatory properties. Zhiyi Xie et al. reviewed how various Chinese herbal polysaccharides, such as those from Atractylodes macrocephala Koidz. and Cordyceps sinensis, bolster immunity by influencing both adaptive and innate responses (64). A systematic review of randomized controlled trials indicates that TCM modulates adaptive immune responses in post-viral fatigue, potentially increasing CD4 T lymphocyte proportions and reducing serum IL-6 levels (65). In addition to regulating humoral immunity, TCM can promote antibody production and enhance immune responses when used as a vaccine adjuvant (66, 67). Moreover, It also helps regulate excessive immune responses and lowers pro-inflammatory factors like IL-6, IL-8, and IL-17A, mitigating systemic inflammation and cytokine storms (56).

Another critical consideration is that immune dysregulation following viral infection is a major driver of severe complications and mortality (20). As the regulation of immune responses is crucial for eliminating pathogens while minimizing immunopathological harm, the targeted modulation of excessive inflammation represents a key therapeutic focus in which TCM may confer substantial benefit. Uncontrolled inflammatory responses, characterized by excessive release of pro-inflammatory cytokines like IL-6 and TNF-α, are primarily driven by persistent activation of signaling pathways such as NF-κB (50). In addition, there is a growing concern regarding TRIM29, which has recently been reported to enhance PERK-mediated endoplasmic reticulum stress immune responses, thereby promoting the production of proinflammatory cytokines (68, 69). These responses are critical in the progression of viral diseases, including influenza and coronaviruses, leading to tissue damage, acute lung injury, multi-organ failure, and increased mortality (20, 21 50). TCM offers notable benefits in managing hyperinflammatory conditions during various stages of viral infections. Isoliquiritigenin, a flavonoid compound, inhibits viral replication and reduces virus-induced inflammation by activating the NRF2 signaling pathway, thereby decreasing oxidative stress and the inflammatory cascade (70). The findings suggest that specific TCM monomers exert targeted regulatory effects on inflammation associated with viral infections. Classical TCM compounds similarly demonstrate notable antiviral and anti-inflammatory properties. Sangju Cold Granule significantly inhibits influenza A virus replication and its related inflammatory response in both *in vitro* and *in vivo* settings. This inhibition is primarily mediated through the suppression of the RIG-I/NF-κB/IFN (I/III) signaling pathway, which is essential for preventing the amplification of cytokine storms (71). Additionally, Qingjin Huatan Decoction decreases IL-6 and TNF-α expression in lung tissues of experimental models, thereby mitigating local inflammatory damage and protecting pulmonary function (72). XiaoEr LianHuaQingGan and Xijiao Dihuang Decoction, widely used for viral infections, significantly reduce fever duration and improve clinical symptoms by suppressing inflammatory mediator production, regulating immune responses, and enhancing mitophagy (73, 74). Chaihu Guizhi Decoction has demonstrated efficacy in diminishing systemic inflammation and modulating immune function in viral diseases (75). Additionally, Liang-Ge-San mitigates lung inflammation by modulating key cytokine levels and reducing neutrophil infiltration in inflamed tissues (76). These findings demonstrate that TCM modulates inflammation and cytokine storms in viral infections through various mechanisms. The regulatory effects on multiple targets and pathways highlight TCM’s unique advantages in preventing and managing virus-induced hyperinflammation and cytokine storms.

Building upon its established capacity to modulate both innate and adaptive immune responses, TCM presents a promising strategy as an immunoadjuvant to augment vaccine-induced immunity and enhance protective efficacy. Vaccines represent a cornerstone of preventive medicine, with their efficacy in mitigating viral diseases, such as polio and hepatitis B, well-documented (77). Adjuvants play a crucial role in shaping the nature and magnitude of the immune response elicited by vaccines (78, 79). In recent years, the application of TCM as vaccine adjuvants has gained increasing attention (67, 80, 81). Polysaccharides derived from TCM plants, including ginseng (82), astragalus (83, 84), Ganoderma lucidum (85, 86), Codonopsis pilosula (87), Rehmannia glutinosa (88), lentinan (89), longan (90), Radix Cyathulae Officinalis (90), and Angelica sinensis (90), have demonstrated significant potential as vaccine adjuvants. The immunomodulatory properties, low toxicity, and favorable safety profiles of these polysaccharides underlie their potential therapeutic applications. They can enhance both humoral and cellular immune responses by activating key immune cells, such as macrophages, T cells, B cells, and NK cells, as well as by regulating cytokine and antibody production (67, 80, 81, 90). Furthermore, these polysaccharides can modulate the intestinal microbiome, promote dendritic cell maturation, and enhance antigen presentation, thereby strengthening both Th1 and Th2 immune pathways (91). Their mechanisms of action also involve interactions with pathogen recognition receptors, including TLRs and NOD-like receptors, which initiate intracellular signaling cascades and immune activation (92, 93). Comprehensive studies have demonstrated the efficacy of these polysaccharides in enhancing vaccine immunogenicity against viral diseases, with minimal adverse effects (83, 84).

In addition to the extensive research on polysaccharides as adjuvants, various active compounds from TCM show significant promise for the development of new vaccine adjuvants. Active components such as saponins (e.g., ginsenosides) (66), flavonoids (e.g., Epimedium flavonoids) (94), tannins and organic acids (e.g., tannic acid) (95), and so on, have been demonstrated to modulate host immune responses, enhance vaccine immunogenicity, and exhibit promising application prospects (67, 96). The multitargeted and immune-balancing properties of TCM adjuvants are anticipated to play a crucial role in enhancing vaccine efficacy, broadening immune coverage, and bolstering public health protection in the future.

Taken together, available evidence suggests that TCM may function as a multifaceted antiviral modality across therapeutic and preventive domains. By concurrently engaging antiviral, anti-inflammatory, and memory-enhancing mechanisms, it offers an integrated approach consonant with the complexity of viral infectious diseases.

### 4.3 Clinical progress

A review of the PubMed database revealed 8 clinical trials examining the use of TCM in modulating immune responses to viral infections. Analysis of these studies highlights key trends and focal areas: (1) The immunomodulatory properties of TCM and natural products in managing viral infections. Clinical evidence suggests that TCM and natural products are increasingly integral to viral infection management, as they modulate inflammatory cytokines, activate immune cell subsets, and facilitate immune recovery. For instance, in a murine model of HCoV-229E infection, Shufeng Jiedu capsules demonstrated significant antiviral and immunoregulatory effects, including reduction of pro-inflammatory cytokines (IL-6, TNF-α, IFN-γ) and elevation of CD4^+^ and CD8^+^ T cell counts (25). Notably, a follow-up clinical real-world study presented in the same publication indicated that Shufeng Jiedu capsules, when administered in conjunction with standard antiviral therapies, facilitated a more rapid resolution of symptoms (such as fatigue and cough) in patients experiencing moderate COVID-19 (25). Additionally, agents such as Qiliqiangxin have demonstrated efficacy in modulating the balance of pro- and anti-inflammatory cytokines (e.g., reducing IFN-γ, IL-17, TNF-α, IL-4 and increasing IL-10) and enhancing cardiac function in viral cardiomyopathy (97). Extracts from Perilla and Portulaca oleracea have been shown to augment NK cell activity and Th1 cytokine production (IL-12, IFN-γ) in healthy individuals (98). (2) Recent advancements in immune reconstitution for chronic viral infections have highlighted innovative strategies to restore immune function. Chinese herbal formulations, including Mianyi granules and the Wenshen Jianpi recipe, have been reported to increase CD4^+^ T cell counts and improve NK cell subpopulations in patients unresponsive to antiretroviral therapy (99, 100). TCM has also been effective in reducing HBV DNA levels and enhancing HBeAg clearance and seroconversion rates in HBeAg-positive chronic hepatitis B patients with normal alanine aminotransferase levels, likely through modulation of the host immune response (101).

### 4.4 Future directions

Despite the demonstrated antiviral, anti-inflammatory, and immunomodulatory effects of various TCM compounds and formulations, their molecular mechanisms remain largely undefined. Current investigations provide only a cursory examination of the interactions between TCM components and viral or host targets, as well as the detailed pathways involved in immune regulation and adjuvant activity. Future research must systematically delineate specific molecular targets and signaling pathways, and explore the relationship between antiviral effects and immune modulation. Advanced structural biology, immunophenotyping, and multi-omics technologies, including transcriptomics, metabolomics, proteomics, and metagenomics, provide robust methodologies to systematically tackle these deficiencies. These methodologies can accurately delineate interaction networks between TCM and host/virus, characterize dynamic immune responses. Moreover, it is essential to prioritize rational integration strategies, such as the combination of TCM with direct-acting antivirals to shorten treatment duration or with vaccines to bolster mucosal immunity, in order to enhance clinical applicability. By employing mechanistic elucidation and evidence-based combination design, TCM can be strategically integrated into contemporary antiviral frameworks, enhancing its contribution to personalized infection management and public health initiatives.

### 4.5 Limitations

This study utilized data from the WoSCC database to offer a comprehensive overview of the research landscape, highlighting major themes, focal points, and emerging trends. This method enhances our comprehension of the field and aids in identifying forthcoming research priorities. Nonetheless, several limitations should be acknowledged. Firstly, relying solely on the WoSCC database may have omitted relevant literature, despite its widespread recognition for quality and suitability for bibliometric analysis. Secondly, the analysis was confined to English-language articles, potentially introducing language bias; however, given English’s predominant position in academia, this limitation is deemed justifiable. Notwithstanding these constraints, the study’s findings are robust and provide valuable insights for guiding future research in this area.

## 5 Conclusion

This study offers the initial comprehensive analysis of worldwide research patterns in utilizing TCM for viral infections through immune modulation in the last decade. It systematically outlines crucial focal points and cutting-edge areas to steer forthcoming research endeavors.

- The publication output in this field has surged twice significantly, aligning with global outbreaks and increased recognition of TCM efficacy against viral infections.
- Global scholars have shown notable interest in research on TCM-mediated immune modulation in viral infections, with China, the United States, India, Iran, and South Korea emerging as the most active nations in this domain, engaging in extensive international research collaborations.
- Noteworthy journals in this area include the Journal of Virology, PLoS One, and Frontiers in Immunology, with the Journal of Virology particularly distinguished for its high citation rate.
- Key research focuses and trends encompass mechanistic investigations into TCM’s antiviral and immunomodulatory effects, TCM’s regulation of inflammation and cytokine storms in viral infections, and TCM’s impact on immune modulation concerning viral infection vaccines.
- Clinical trials in this field concentrate on refining viral infection management strategies by leveraging the immunomodulatory effects of TCM and natural products, as well as exploring innovative methods for immune reconstitution in chronic viral infections.

In conclusion, this research provides valuable insights into the current trends and focal points of TCM in addressing viral infections through immune modulation. It offers essential context and precise direction for forthcoming innovative research endeavors.

## Data Availability

The original contributions presented in the study are included in the article/**Supplementary Material**. Further inquiries can be directed to the corresponding authors.
