# Supplementary material for: Bibliometric analysis of traditional Chinese medicine for viral infections through immune modulation (2015–2025)
Source: Front Immunol. 2025 Sep 26;16:1647900. doi: 10.3389/fimmu.2025.1647900 (PMC12510926; doi:10.3389/fimmu.2025.1647900)
Supplement: Supplementary file 1 [file Table1.docx]

**Web of Science database search formula**

((((TS=("Viral Infection*" OR "Viral Disease*" OR virus OR viruses ) AND TS=("Immunomodulation*" OR "Immunomodulatory Therapy*" OR "Immunity" OR "Immune" OR "Immunotherapy" OR "Immunostimulat*" OR "Immunosuppress*") AND TS=("Chinese medicine" OR TCM OR herbal OR extract OR "botanical medicine" OR "Compound Prescription" OR "material medicine" OR decoction OR tang OR powder OR san OR pill OR "Chinese proprietary medicine" OR "Chinese patent medicine" OR granules OR plaster* OR "oral liquid*" OR gels OR capsule OR syrup)) AND DOP=(2015-05-04/2025-05-04)) AND DT=(Article OR Review)) AND LA=(English)(Note: TS=Topics; DOP=Publication date; DT=Document type; LA=Language).

**PubMed database search formula**

((("Viral Infection*"[Title/Abstract] OR "Viral Disease*"[Title/Abstract] OR virus[Title/Abstract] OR viruses[Title/Abstract]) AND ("Immunomodulation*"[Title/Abstract] OR "Immunomodulatory Therapy*"[Title/Abstract] OR "Immunity"[Title/Abstract] OR "Immune"[Title/Abstract] OR "Immunotherapy"[Title/Abstract] OR "Immunostimulat*"[Title/Abstract] OR "Immunosuppress*"[Title/Abstract])) AND ("Chinese medicine"[Title/Abstract] OR TCM[Title/Abstract] OR herbal[Title/Abstract] OR extract[Title/Abstract] OR "botanical medicine"[Title/Abstract] OR "Compound Prescription"[Title/Abstract] OR "material medicine"[Title/Abstract] OR decoction[Title/Abstract] OR tang[Title/Abstract] OR powder[Title/Abstract] OR san[Title/Abstract] OR pill[Title/Abstract] OR "Chinese proprietary medicine"[Title/Abstract] OR "Chinese patent medicine"[Title/Abstract] OR granules[Title/Abstract] OR plaster*[Title/Abstract] OR "oral liquid*"[Title/Abstract] OR gels[Title/Abstract] OR capsule[Title/Abstract] OR syrup[Title/Abstract])) AND (("2015/05/04"[Date - Publication] : "2025/05/04"[Date - Publication])) Filters: Clinical Trial
