# Supplementary material for: Bibliometric analysis of traditional Chinese medicine for viral infections through immune modulation (2015–2025)
Source: Front Immunol. 2025 Sep 26;16:1647900. doi: 10.3389/fimmu.2025.1647900 (PMC12510926; doi:10.3389/fimmu.2025.1647900)
Supplement: Supplementary file 5 [file Table5.docx]

Clinical Trials on Influenza Co-Infections

| Number | Publication year | Title |
| --- | --- | --- |
| 1 | 2016 | Effect of Rorrico, extracted from group of Chinese medicines, on influenza A and H1N1 infections. |
| 2 | 2018 | Comparative Proteomic Profiling and Biomarker Identification of Traditional Chinese Medicine-Based HIV/AIDS Syndromes. |
| 3 | 2020 | Efficacy of a Chinese herbal formula on hepatitis B e antigen-positive chronic hepatitis B patients. |
| 4 | 2021 | Shufeng Jiedu, a promising herbal therapy for moderate COVID-19:Antiviral and anti-inflammatory properties, pathways of bioactive compounds, and a clinical real-world pragmatic study |
| 5 | 2022 | Efficacy and safety of Mianyi granules (+mianyi+) for reversal of immune nonresponse following antiretroviral therapy of human immunodeficiency virus-1: a randomized, double-blind, multi-center, placebo-controlled trial. |
| 6 | 2022 | Wenshen Jianpi recipe induced immune reconstruction and redistribution of natural killer cell subsets in immunological non-responders of human immunodeficiency virus/acquired immune deficiency syndrome: a randomized controlled trial. |
| 7 | 2024 | Qiliqiangxin Alleviates Imbalance of Inflammatory Cytokines in Patients with Dilated Cardiomyopathy: A Randomized Controlled Trial. |
| 8 | 2025 | Immunomodulatory effects of supercritical CO(2) extracted oils from Portulaca oleracea and Perilla frutescens (PPCE) in healthy individuals: a randomized double-blind clinical trial. |
